# Early Developmental Exposure to General Anesthetic Agents in Primary Neuron Culture Disrupts Synapse Formation via Actions on the mTOR Pathway

**DOI:** 10.3390/ijms19082183

**Published:** 2018-07-26

**Authors:** Jing Xu, R. Paige Mathena, Michael Xu, YuChia Wang, CheJui Chang, Yiwen Fang, Pengbo Zhang, C. David Mintz

**Affiliations:** 1 Department of Anesthesiology and Critical Care Medicine, The Johns Hopkins University School of Medicine, Baltimore, MD 21205, USA; jxu72@jhmi.edu (J.X.); rmathen1@jhmi.edu (R.P.M.); michael.xu@downstate.edu (M.X.); yw3af@virginia.edu (Y.W.); zhpbo@mail.xjtu.edu.cn (P.Z.); 2 Chang Gung Memorial Hospital, Taoyuan City 33305, Taiwan; jeff83831@gmail.com (C.C.); gs1606890220@gmail.com (Y.F.)

**Keywords:** anesthesia, neurotoxicity, synapse, mTOR, neurodevelopment

## Abstract

Human epidemiologic studies and laboratory investigations in animal models suggest that exposure to general anesthetic agents (GAs) have harmful effects on brain development. The mechanism underlying this putative iatrogenic condition is not clear and there are currently no accepted strategies for prophylaxis or treatment. Recent evidence suggests that anesthetics might cause persistent deficits in synaptogenesis by disrupting key events in neurodevelopment. Using an in vitro model consisting of dissociated primary cultured mouse neurons, we demonstrate abnormal pre- and post-synaptic marker expression after a clinically-relevant isoflurane anesthesia exposure is conducted during neuron development. We find that pharmacologic inhibition of the mechanistic target of rapamycin (mTOR) pathway can reverse the observed changes. Isoflurane exposure increases expression of phospho-S6, a marker of mTOR pathway activity, in a concentration-dependent fashion and this effect occurs throughout neuronal development. The mTOR 1 complex (mTORC1) and the mTOR 2 complex (mTORC2) branches of the pathway are both activated by isoflurane exposure and this is reversible with branch-specific inhibitors. Upregulation of mTOR is also seen with sevoflurane and propofol exposure, suggesting that this mechanism of developmental anesthetic neurotoxicity may occur with all the commonly used GAs in pediatric practice. We conclude that GAs disrupt the development of neurons during development by activating a well-defined neurodevelopmental disease pathway and that this phenotype can be reversed by pharmacologic inhibition.

## 1. Introduction

The United States Food and Drug Administration has recently required that 12 commonly used anesthetic and sedative agents with mechanisms of action on NMDA and GABA receptors carry labels warning that repeated or lengthy exposure to these drugs between the third trimester and the first three years of life may result in adverse consequences for brain development (FDA Drug Safety Communication). An estimated 115,000 children each year are anesthetized for surgery and other procedures in the U.S. alone, suggesting that millions of children are exposed to anesthesia each year worldwide [1]. It is not yet clear which patients are potentially at risk of cognitive dysfunction related to this exposure, but early results from the only two clinical trials that have reached endpoints give reassurance that short, single exposures in healthy children do not have deleterious effects [2,3]. This finding is consistent with data from large epidemiologic studies showing no effect of short, single early life exposures to surgery and anesthesia, but a correlation between long or multiple exposures and reduced scores on cognitive testing, worsened scores in educational testing assessments, and increased billing codes indicates developmental or behavioral disorders [4,5,6]. Numerous studies have found that early postnatal exposure to GA in rodents results in deficits in performance on tests of learning and memory [7,8,9,10,11,12,13,14,15], but rodent anesthesia models introduce a confound of physiologic perturbation that is difficult to measure and also the short timeline of rodent brain development might exaggerate the consequences of a toxic developmental exposure. However, recent data in non-human primates have provided definitive evidence that early postnatal GA exposure can have lasting effects on cognition, including deficits in socioemotional and learning function [16,17,18,19].

The mechanism by which a transient exposure to GA could have lasting consequences on brain development has been the subject of considerable investigation, but no clear conclusion has been reached [20,21]. We have found evidences in an in vivo mouse model that early postnatal exposure to isoflurane causes a lasting increase in activity in the mTOR pathway in the hippocampal dentate gyrus. Inhibition of mTOR upregulation with rapamycin reversed a loss of neuronal spines in dentate gyrus granule neurons and also restored performance on hippocampal-dependent learning tests that are impaired by isoflurane exposure [8]. The mTOR pathway is a complex and heterogeneous signaling system that integrates intra- and extracellular cue sensing and links to numerous other signaling pathways in order to regulate metabolism, growth, and homeostasis [22]. A lasting anesthetic action on mTOR function is an intriguing potential mechanism of developmental anesthetic neurotoxicity. The mTOR system is critical for neuronal development [23] and a causative role of mTOR system dysfunction has been proposed for better understood neurodevelopmental disorders, such as Fragile X, autism, schizophrenia, and drug addiction [24]. However, mTOR has not been extensively studied in this context, and the evidence linking it to anesthetic toxicity is mixed [25].

Here we use an in vitro primary rat neuron culture system to further explore the hypothesis that GAs disrupt neuron development via an upregulation of mTOR signaling. To this end we employ quantitative immunohistochemistry to examine the effects of anesthetic-induced mTOR changes on synapse development. We also test for contributions of the mTOR1 and mTOR2 complexes, which represent a divergence in the pathway. Finally, we ask whether the effects on the mTOR pathway generalize to multiple anesthetic agents.

## 2. Results and Discussion

### 2.1. Effects of Isoflurane Exposure for 6 h on Apoptosis

**Figure 1:**
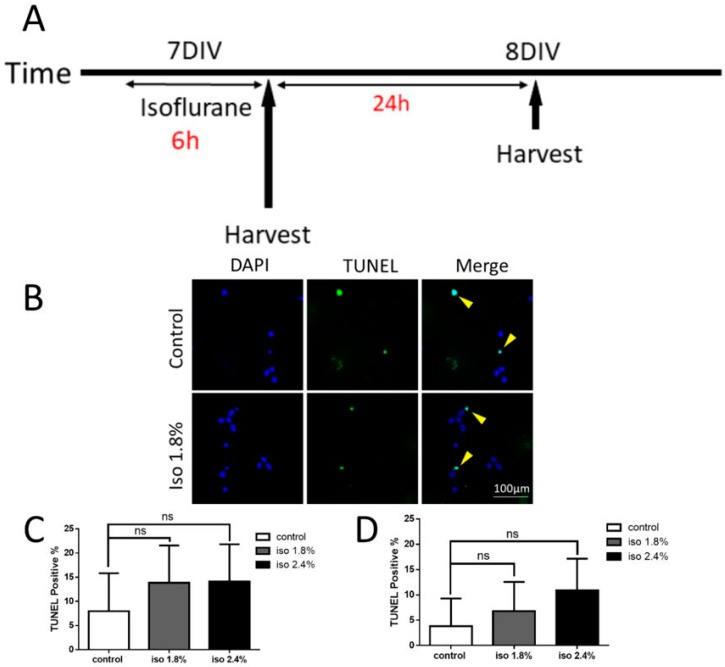
Isoflurane exposure at different doses did not show any significant apoptosis in our exposure paradigms. (**A**) The TUNEL experiment timeline in vitro. The neurons were exposed to either carrier gas or 1.8%/2.4% isoflurane for 6 h on their 7 DIV, and the cells were harvested immediately after exposure or 24 h after the exposure; (**B**). Representative images of DAPI (blue) and TUNEL (green) immunofluorescence in the dissociated neurons at 8 DIV; (**C**,**D**) 6 h of isoflurane treatment on 7 DIV did not show a significant apoptosis difference among all the DAPI/TUNEL neurons compared to the control groups immediately after the exposure (**C**) or 24 h after the exposure (**D**) (*n* = 15 fields that were measured per group, n.s. indicates no significant difference, one-way ANOVA with Dunnett’s multiple comparisons test).

In order to exclude the possible effects of isoflurane on the health of the primary neurons, we first tested the difference in apoptosis in the 1.8% and 2.4% isoflurane exposure group compared to the control group. A small number of cells stained positive in the TUNEL assay following a 6 h exposure both when the cells were harvested immediately after exposure and when they were harvested 24 h after exposure (Figure 1B). There was no significant difference between the control group (7.95 ± 7.86%) and the isoflurane 1.8% (13.85 ± 7.69%) and 2.4% (14.14 ± 7.67%) groups when harvested directly after exposure using one-way ANOVA with Dunnett’s multiple comparisons (Figure 1C). The same results were found when the cells were harvested 24 h following exposure ((control group (3.83 ± 5.47%), isoflurane 1.8% (6.81 ± 5.77%), and 2.4% (10.91 ± 6.25%) groups)) (Figure 1D). These results indicate that 1.8% and 2.4% isoflurane exposure on seven days in vitro (DIV) for 6 h does not affect normal baseline levels of cell apoptosis.

### 2.2. Effects of 1.8% Isoflurane Exposure for 6 h on Synaptogenesis

**Figure 2:**
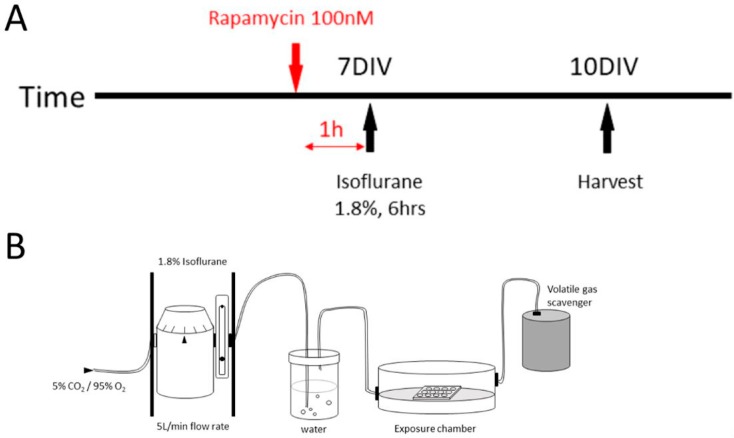
Schematic representation of the experimental timeline and exposure induction diagram in vitro. (**A**) The general experiment timeline in vitro. The neurons were exposed to 1.8% isoflurane for 6 h on their 7 DIV, and 100 nM rapamycin was added into the media 1 h before the exposure according to the experiment design. The fresh media change was done regularly. The cells were fixed for immunohistochemistry on 10 DIV; (**B**) Coverslips in 12-well plates were placed in identical air-tight, humidified chambers. Isoflurane was delivered using an agent-specific, calibrated inline and was diluted in 5% CO_2_/95% O_2_ carrier gas. Controls for these experiments received 5% CO_2_/95% O_2_ carrier gas only. After a 15-min equilibration period, the sealed chambers were placed in an incubator to maintain a temperature at 37 °C for the duration of the anesthesia exposure.

**Figure 3:**
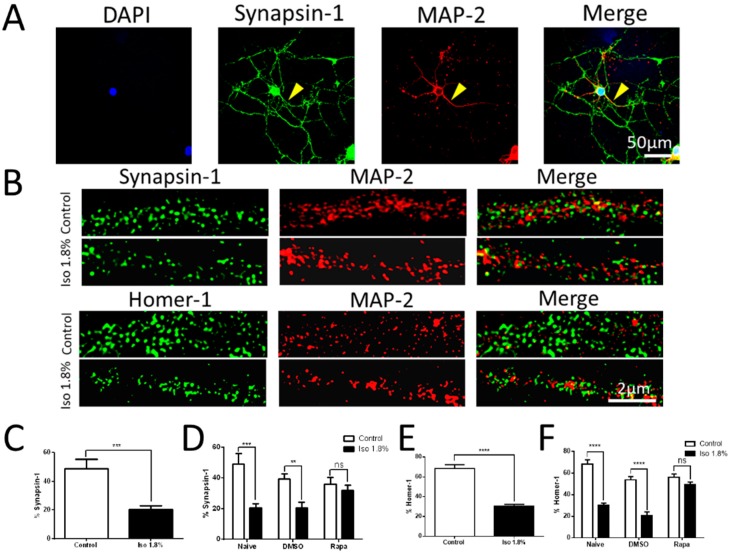
A 1.8% isoflurane exposure for 6 h decreases pre- and post-synaptic marker intensity in vitro. (**A**,**B**) Representative images of Synapsin-1/Homer-1 (green), MAP-2 (red), DAPI (blue) immunofluorescence in neurons in dissociated culture at 10 DIV are shown. The segment for the dendrite was picked according to MAP-2 staining from each neuron and the locations for images taken were defined as 20–30 μm from the nucleus according to DAPI (shown as the yellow arrow pointing in (**A**); (**C**–**F**) 6 h of isoflurane exposure on 7 DIV caused a significant difference in the intensity decrease of Synapsin-1 compared to the control group (**C**), while rapamycin treatment before the isoflurane exposure reversed the Synapsin-1 intensity to normal compared to the control with rapamycin treatment group (**D**). The intensity of Homer-1 also decreased compared to the control group (**E**), while rapamycin treatment before the isoflurane exposure reversed the Homer-1 intensity to normal compared to the control with rapamycin treatment group (**F**). (*n* = 30 neurons measured per group, ** *p* < 0.01, *** *p* < 0.001, **** *p* < 0.0001, n.s. indicates no significant difference, *t*-test for (**C**,**E)**, two-way ANOVA with Bonferroni’s multiple comparisons test for (**D**,**F**).

Our previous work in newborn dentate gyrus granule neurons in the intact mouse showed that isoflurane could act via an mTOR-mediated mechanism to cause a lasting reduction in the numbers of dendritic spines, which represent a morphological marker for excitatory post-synaptic elements. To determine whether this effect is an acute one that occurs during neuron synapse development and to test whether it generalizes to multiple neuronal types, we explored the effects of isoflurane administered during the period of ongoing synaptogenesis in cultured neocortical neurons, a population that is both heterogeneous and distinctly different from dentate gyrus neurons. Exposures consisting of 1.8% isoflurane for 6 h were performed at 7 DIV when synaptogenesis is ongoing, and results were assayed at 10 DIV when it is largely complete [26] (Figure 2). Double immunofluorescence staining was performed using MAP-2 as a dendritic marker to define the area over which synaptic markers were measured, and either Synapsin-1 to identify pre-synaptic elements or Homer-1 to identify excitatory post-synaptic elements. The locations of the images taken for analysis were 50 μm from the nucleus, representative images are shown in Figure 3A (scale bar: 50 µm) and Figure 3B (scale bar: 2 µm).

We found that 6 h of isoflurane treatment at a concentration of 1.8% resulted in a significant decrease in the intensity of Synapsin-1 immunoreactivity (20.46 ± 7.33%) compared to the control group (48.95 ± 19.02%, *p* < 0.001) (Figure 3C). Rapamycin treatment results in Synapsin-1 intensity levels (32.11 ± 9.10%) that are not significantly different from the control plus rapamycin treatment group (36.13 ± 11.70%), while there was a significant interaction of isoflurane treatment and rapamycin using two-way ANOVA with Bonferroni’s multiple comparisons test, suggesting a rescue effect of rapamycin (Figure 3D). Carrier gas and isoflurane treatment were also used in the presence of the rapamycin diluent, dimethyl sulfoxide (DMSO), and the results did not differ from the same experiment performed without DMSO, indicating that the diluent has no independent effect. Isoflurane treatment at 1.8% for 6 h resulted in a significant reduction in intensity of Homer-1 immunoreactivity (30.47 ± 5.22%) compared to the control group (68.46 ± 11.18%, *p* < 0.0001) (Figure 3E). As was found with Synapsin-1, rapamycin treatment after isoflurane exposure prevented the effects of isoflurane. Homer-1 immunoreactivity after rapamycin treatment did not differ significantly between the isoflurane (49.33 ± 7.32%) and carrier gas groups (56.14 ± 8.91%) (Figure 3F). Taken together, these data indicate that isoflurane interferes with the formation of excitatory synapses in developing cultured neocortical neurons and that this effect may be due to actions on the mTOR pathway.

Our own work in vivo shows that newborn dentate gyrus neurons in mice exposed to GA with isoflurane are found to have reduced numbers of spines overall, and profoundly-reduced numbers of mushroom morphology spines over a month later [8]. As in our culture model, we found that this effect was reversible by treatment with rapamycin not acutely, but for a week after the exposure. While dendritic spines are generally the sites of excitatory post-synaptic elements, the correlation is imperfect, and our finding of reduced Homer-1 immunoreactivity in culture lends weight to our previous findings in vivo, particularly as we also found a decrease in expression of a pre-synaptic marker as well. However, our results in this manuscript differ in some important ways. Our anesthesia exposure occurred during synaptogenesis, rather than at the point of generation, and also the neurons observed in a cortical culture differ morphologically and functionally from dentate gyrus granule cells, which have many unusual features compared to other neurons. Thus, we predict, based on our findings, that mTOR-mediated effects on synapse formation are likely to generalize across a broad range of contexts. The current literature does not have any other studies of mTOR and anesthetic effects on synapses, but the preponderance of evidence suggests at least that GA exposure during development can disrupt synapse formation or maintenance. Two in vivo rodent studies using electron microscopy to identify synapses found decreased synaptic density in the hippocampus of young adult mice that had been exposed to GAs during the early postnatal phase [27,28]. Interestingly, when this phenomenon was studied in the rodent pre-frontal cortex using light microscopy to quantify spine numbers, it was found that a P5 exposure reduced spine number but a P15 exposure actually increased the spine number [29], suggesting that the state of the neuron at the time of exposure is critically important to determine the effect of GAs. Our findings in this manuscript support the conclusion that GA exposure prior to stabilization of synapses leads to a failure of synapse formation.

### 2.3. Parameters of Activation of mTOR by Isoflurane in Cultured Neurons

**Figure 4:**
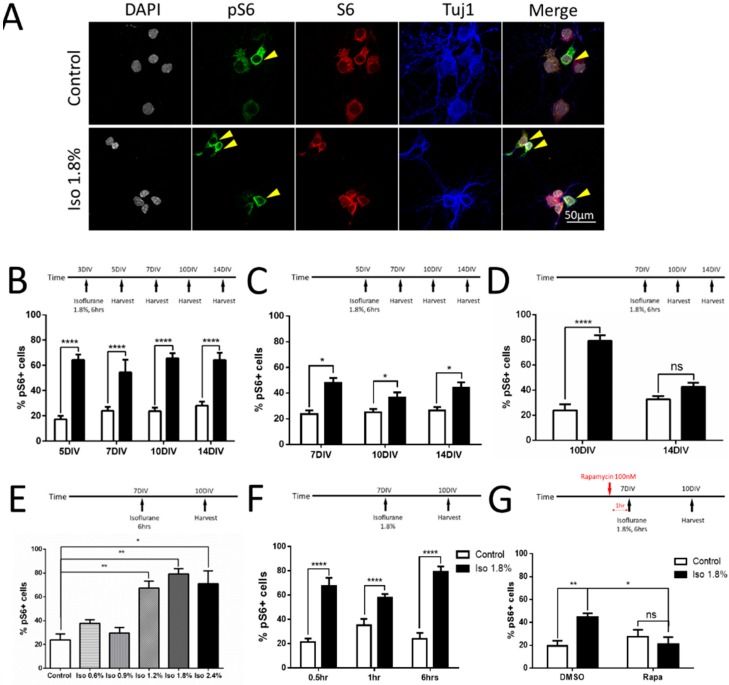
Isoflurane exposure at different time points showed effects on the downstream marker of mTOR pathway. (**A**) Representative images of DAPI (grey), pS6 (green), S6 (red), βIII Tubulin (blue) immunofluorescence in the dissociated neurons at 10 DIV. (**B**–**G**) 6 h of 1.8% isoflurane treatment on different early time points caused significant increases in the percentage of pS6 positive cells among all the DAPI/Tubulin neurons compared to the control group at late time points except the ones exposed on 7 DIV and tested on 14 DIV (**B**–**D**). The effect on pS6 levels at 10 DIV varied depending on the doses of isoflurane. There was a significant increase in immunoactivity starting from the 1.2% isoflurane group to the 2.4% isoflurane group, while lower doses (0.6% and 0.9%) remained at control levels of pS6 immunoactivity (**E**). Different exposure durations (0.5 h, 1 h and 6 h) of 1.8% isoflurane also resulted in increased pS6 immunoactivity at all exposure times compared to control (**F**). Adding rapamycin, an mTOR pathway inhibitor, reversed the increase of pS6 after isoflurane exposure on 7 DIV (**G**). (*n* = 15 fields that were measured per group, * *p* < 0.05, ** *p* < 0.01, **** *p* < 0.0001, n.s. indicates no significant difference, *t*-test for (**B**–**F**). one-way ANOVA with Dunnett’s multiple comparisons test for (**E**), two-way ANOVA with Bonferroni’s multiple comparisons test for (**G**).

We have previously shown that isoflurane exposure causes a lasting increase in expression of phospho-S6 (pS6), a commonly used marker of activity in the mTOR pathway [8,25]. However, the constraints of in vivo experimentation are such that we were unable to determine at what stage of development neurons are subject to this phenomenon, and we were also unable to test the minimum time of exposure and exposure dose required. To address these questions, we stained for DAPI (grey) to define cell bodies and immunolabeled for βIII-tubulin (blue) to verify neuronal cell type. To measure the activity in the mTOR pathway, we co-labeled for unphosphorylated-S6 (red) and phosphorylated-S6 (green) to assess mTOR activation. A representative example of control and isoflurane 1.8% for 6 h treatment on 7 DIV with harvest on 10 DIV is shown (Figure 4A, scale bar: 50 µm).

We first tested the effects of varying the time of exposure to isoflurane on pS6 expression. We found that 6 h of 1.8% isoflurane treatment on 3 DIV caused a significant increase in the percentage of pS6 positive neurons (as the yellow arrows pointed out in Figure 4A) compared to the control group with harvest at 5 DIV (64.25 ± 15.95% vs. 17.22 ± 10.15%, *p* < 0.0001), 7 DIV (54.33 ± 37.69% vs. 23.98 ± 11.54%, *p* < 0.0001), 10 DIV (65.53 ± 15.26% vs. 23.73 ± 9.60%, *p* < 0.0001), and 14 DIV (64.17 ± 21.40% vs. 28.01 ± 11.92%, *p* < 0.0001) (Figure 4B). Isoflurane treatment at 5 DIV caused a significant increase in the percentage of pS6+ neurons compared to the control group at 7 DIV (48.00 ± 11.43% vs. 23.60 ± 11.33%, *p* < 0.05), 10 DIV (36.65 ± 14.74% vs. 25.13 ± 9.63%, *p* < 0.05), and 14 DIV (44.36 ± 15.36% vs. 26.65 ± 9.57%, *p* < 0.05) (Figure 4C). Exposure at 7 DIV caused a significant increase in pS6 positive neurons compared to the control group on 10 DIV (79.21 ± 16.54% vs. 23.86 ± 18.39%, *p* < 0.0001), but no difference was detected at the 14 DIV (42.51 ± 12.51% vs. 32.74 ± 7.70%) harvest time point (Figure 4D). These findings suggest that isoflurane exposure causes pS6 to increase at any early developmental time point, but that the effect is reduced as the neuron approaches maturity.

Next, we tested the effects of different concentrations of isoflurane delivered at 7 DIV and assayed for pS6 on 10 DIV. There was a significant difference between the 1.2% isoflurane group (67.33 ± 22.31%, ANOVA, *p* < 0.01), 1.8% isoflurane group (79.20 ± 16.53%, ANOVA, *p* < 0.01), and 2.4% isoflurane group (71 ± 32.31%, ANOVA, *p* < 0.05), compared to the control group (23.86 ± 18.39%), while there was no significant difference between the 0.6% isoflurane group (37.80 ± 11.13%), 0.9% isoflurane group (29.65 ± 13.18%), and the control group using one-way ANOVA with Dunnett’s multiple comparisons (Figure 4E). This represents a clear inflection point at a value corresponding to one adult minimum alveolar concentration (MAC), which is a clinically-reasonable dose in pediatric settings.

We then sought to determine the minimum duration of exposure to isoflurane that is required to cause an increase in mTOR signaling. We exposed P7 neurons to 1.8% isoflurane with varying durations and measured pS6 levels on 10 DIV. There was a significant difference between the 0.5 h isoflurane group (67.28 ± 26.06%) compared to the control group (21.40 ± 10.43%, *p* < 0.0001), 1 h isoflurane group (58.00 ± 10.62%) compared to the control group (35.07 ± 19.39%, *p* < 0.0001), and 6 h isoflurane group (79.20 ± 16.53%) compared to the control group (23.86 ± 18.39%, *p* < 0.0001) (Figure 4F). Half an hour exposure is the shortest practical duration to measure in our model, and we conclude that even brief exposures have the potential to act on the mTOR pathway.

In order to further confirm that the increase in pS6 labeling that we observe is, in fact, evidence of mTOR pathway activation, we treated the cultures with rapamycin as in Figure 1. With the significant interaction between isoflurane and rapamycin treatment using two-way ANOVA with Bonferroni’s multiple comparisons test, we found that there was a significant increase in the percentage of pS6 positive cells among all the DAPI/Tubulin neurons between the isoflurane + vehicle (DMSO) group (44.49 ± 9.73%) compared to the control + vehicle (DMSO) group (19.44 ± 16.86%, *p* < 0.01). Rapamycin treatment prevented the increase of pS6 immunoactivity in the isoflurane group (21.00 ± 23.25%) compared to the isoflurane group without rapamycin (44.49 ± 9.73%, *p* < 0.05), and there was no significant difference between isoflurane + rapamycin group compared to the control+ rapamycin group (27.52 ± 23.06%) (Figure 4G).

The use of a dissociated culture model presents a substantial advantage for studying the pharmacology of anesthetic toxicity as compared to in vivo models, as the short timeline of experiments and the lesser requirements for resources allow for the study of a broad range of doses and exposure paradigms. The general consensus in the literature is that the period of synaptogenesis represents the peak window of vulnerability to developmental anesthetic neurotoxicity in vivo [30,31], but in vivo synaptogenesis is a heterogeneous process that occurs over long periods of time as different cohorts of neurons mature over widely variable timelines. Using the culture model, in which synaptogenesis is synchronous starting from 5 DIV and ending about 14 DIV [32], we asked which stages of synaptogenesis are vulnerable to a potentially harmful increase in the mTOR pathway in response to isoflurane exposure to gain a clearer understanding of the potential window of vulnerability. The only time point we studied at which pS6 up-regulation due to isoflurane exposure was at all abated was the P7 exposure with measurement of pS6 at 14 DIV. Synapses are highly dependent on filamentous actin for stability during the first week in culture, but during the second week there is a marked shift towards persistence of synapses even when actin is perturbed [33]. Several previous studies have suggested that isoflurane toxicity during development may be mediated in part via effects on the actin cytoskeleton [34,35], and our results are consistent with the period of actin-dependent synapse formation as the window of vulnerability to the mTOR-mediated effects on synaptogenesis. One of the principal concerns in the study of developmental anesthetic toxicity is that many reported phenomena may lack clinical relevance as they are reported by studies that use only supra-therapeutic doses, sometimes in excess of 2 adult MAC, or unrealistically long exposure times, which in some cases are as much as 24 h [36]. Our findings in cultured neurons show that the vulnerability of neurons to isoflurane-induced mTOR activation appears to have a threshold between 0.9% and 1.2% isoflurane, which is a dose that is clinically realistic as it represents less than 1 MAC for pediatric patients [37]. Furthermore, the duration of exposure required to generate a significant effect is strikingly short at 30 min, the briefest exposure that is practical in our system. This finding does call into question the clinical relevance of mTOR activation as the evidence from clinical trials suggests that anesthetic exposures under an hour do not have measurable effects on children [38,39]. However, it is reasonable to suppose that, in vivo, particularly in the setting of a complex brain with a long developmental timeline, there may be a high threshold for phenotypically detectable events, which exceeds the threshold for detectable change at the cellular and molecular levels. Nevertheless, the discrepancy between thresholds of toxicity in rodent models, humans, and non-human primates remains an unsolved problem in the field of anesthetic toxicity in neuro-development [40].

### 2.4. Effects of Isoflurane Exposure on the mTORC1 and mTORC2 Pathway

**Figure 5:**
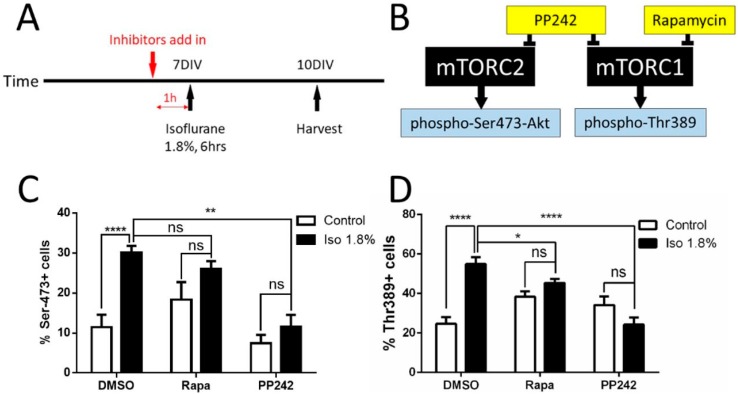
Effects of 1.8% isoflurane exposure for 6 h on the downstream marker of mTORC1 and mTORC2 pathway. (**A**) The timeline for adding mTORC1/mTORC2 inhibitors. The inhibitors were added to the media 1 h before the 1.8% isoflurane/carrier gas exposure on 7 DIV. The cells were fixed for immunohistochemistry on 10 DIV; (**B**). Visual diagram showing the inhibition of PP242 and rapamycin on mTORC1 and mTORC2 pathways, (**C**,**D**) For the mTORC2 downstream marker Ser473-Akt, there is a significant increase after 1.8% isoflurane exposure for 6 h on 7 DIV compared to the control group. Adding rapamycin did not fully reverse it back to normal, but adding PP242 made a significant difference between the isoflurane + PP242 and isoflurane + DMSO groups, while the positive Ser-473 cells returned back to normal compared to the control + PP242 group. This indicates that the mTORC2 pathway is involved in the isoflurane neurotoxicity changes (**C**). For the mTORC1 downstream marker Thr389, isoflurane exposure increased its immunoactivity significantly, while adding either rapamycin or PP242 reversed its immunoactivity back to normal. This indicates that mTORC1 pathway is also involved in the deficiency of neuron growth caused by isoflurane as well (**D**). (*n* = 15 fields that were measured per group, * *p* < 0.05, ** *p* < 0.01, **** *p* < 0.0001, n.s. indicates no significant difference, two-way ANOVA with Bonferroni’s multiple comparisons test for (**C**,**D**).

The mTOR pathway has two principal branches, which arise from mTORC1 and mTORC2. These pathways perform biologically distinct functions in some settings, but there is substantial communication between them [41]. We next sought to determine whether the effects of isoflurane are mediated through one branch of the pathway. This was accomplished via a series of experiments using mTOR pathway inhibitors with differential effects between mTORC1 and mTORC2, and by measuring levels of immunoreactivity of downstream phospho-proteins that are activated differentially between the pathway branches. Inhibitors were added into the media one hour before the 1.8% isoflurane/carrier gas exposure at 7 DIV for a harvest at 10 DIV (Figure 5A). The concentrations of the inhibitor were maintained after the exposure by media change with fresh inhibitor on 8 DIV and 9 DIV. The branch specific inhibitor and readout strategy (shown in Figure 4B) is as follows: PP242 was used as an inhibitor to block both mTORC1 and mTORC2 pathways simultaneously. Rapamycin was used as an mTORC1-specific pathway inhibitor. Ser473 phosphorylated Akt (pAkt, Ser473) was used as an mTORC2 downstream activity marker, while Thr389 phosphorylated 70S6 (p70S6, Thr389) was used as an activity marker downstream from mTORC1 (Figure 5B). The combination of these inhibitors and markers has been shown to be effective in differentiating activity in between the mTORC1 and mTORC2 branches [42].

We found a significant difference in the percentage of pAkt positive neurons between the isoflurane + vehicle (DMSO) group (30.19 ± 6.12%) and the control + vehicle (DMSO) group (11.45 ± 11.71%, *p* < 0.0001). As expected, with the significant interaction between isoflurane and inhibitor treatments using two-way ANOVA with Bonferroni’s multiple comparisons test, rapamycin treatment did not change the pAkt levels which were shown in the isoflurane + rapamycin group (26.09 ± 7.04%) compared to the isoflurane + DMSO group, but there was a significant difference between the isoflurane + PP242 group (14.60± 14.50%) compared to the isoflurane + DMSO group (*p* < 0.01). A comparison between the isoflurane + PP242 group and the control + PP242 group (4.16 ± 5.27%) showed no significant difference (Figure 5C). Taken together, this data suggests that the mTORC2 pathway is involved in the toxic effect of isoflurane on neurons. There was a significant increase in the percentage of Thr-389 positive cells among all the DAPI/Tubulin neurons between the isoflurane + vehicle (DMSO) group (54.88 ± 10.56%) compared to those of the control + vehicle (DMSO) group (24.67 ± 10.19%, *p* < 0.0001) while there was a significant interaction between isoflurane and inhibitor treatments using two-way ANOVA with Bonferroni’s multiple comparisons test (Figure 4D). Adding rapamycin before the exposure prevented the changes in Thr-389 levels (45.37 ± 6.09%) seen with the isoflurane + DMSO group (*p* < 0.05), and there was a significant difference between the isoflurane + PP242 group (24.22 ± 13.66%) compared to the isoflurane + DMSO group (*p* < 0.0001). Comparison between the isoflurane + PP242 group and the control + PP242 group (34.15 ± 16.55%) showed no significant difference (Figure 5D). Taken together, these data indicate that isoflurane acts on both the mTORC1 and mTORC2 branches. This is principally significant because it shows that therapeutic strategies cannot be designed around only one pathway branch or the other, unless it can be determined that the deleterious effects occur downstream of only one of the two branches.

### 2.5. Effects of Sevoflurane and Propofol on the Downstream Marker of the mTOR Pathway

A key question in developmental anesthesia toxicity is whether unwanted effects of anesthetic agents could be avoided through different choices of the primary anesthetic drug used. Thus, we asked what the effects of sevoflurane, the most commonly used volatile agent in pediatric anesthesia practice, and propofol, which is an intravenous agent that serves as the next likely alternative to isoflurane or sevoflurane, are on the mTOR pathway. Sevoflurane exposure in cultured neurons was accomplished using the same methods used for isoflurane exposure. Propofol exposure was accomplished by adding propofol in a carrier to the culture media, followed by media replacement at the appropriate time to terminate the exposure.

**Figure 6:**
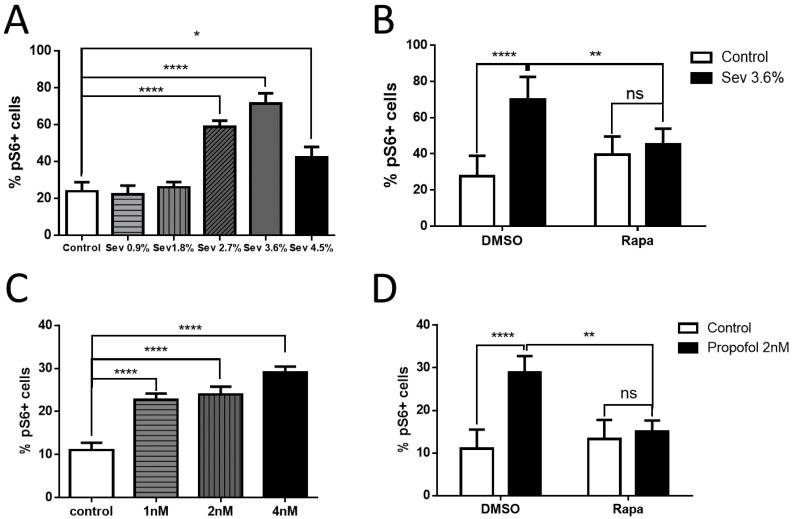
Effects of sevoflurane and propofol on the downstream marker of the mTOR pathway. (**A**,**B**) The effect on pS6 levels at 10 DIV varied depending on the doses of sevoflurane at 7 DIV. There was a significant increase in immunoactivity starting from the 2.7% sevoflurane group to the 4.5% sevoflurane group, while lower doses (0.9% and 1.8%) remained at control levels of pS6 (**A**). Rapamycin treatment prevented the increase in pS6 labeling with 3.6% sevoflurane exposure (**B**). (**C**,**D**) Different doses of propofol at 7 DIV had similar effects on pS6 levels at 10 DIV. There was a significant increase in pS6 immunoactivity starting from the 1 nM propofol group to the 4 nM propofol group (**C**). Rapamycin treatment prevented the increase in pS6 labeling with 2 nM propofol exposure (**D**). (*n* = 15 fields that were measured per group, * *p* < 0.05, ** *p* < 0.01, **** *p* < 0.0001, n.s. indicates no significant difference, one-way ANOVA with Dunnett’s multiple comparisons test for (**A**,**C**), two-way ANOVA with Bonferroni’s multiple comparisons test for (**B**,**D**).

We measured the effect of a range of clinically relevant concentrations of sevoflurane and propofol delivered at 7 DIV on pS6 levels measures at 10 DIV. Using one-way ANOVA with Dunnett’s multiple comparisons test, we found no significant difference in the percentage of neurons positive for pS6 between the 0.9% sevoflurane group (22.29 ± 14.86%) or the 1.8% sevoflurane group (26.03 ± 10.52%) and the control group (23.85 ± 18.39%) (Figure 6A). However, at 2.7% sevoflurane (59.00 ± 12.11%, *p* < 0.0001), 3.6% sevoflurane (71.35 ± 21.27%, *p* < 0.0001), and 4.5% sevoflurane group (42.39 ± 20.91%, *p* < 0.05), there was a significant increase in the percentage of pS6+ neurons over control (Figure 5A). With the significant interaction between isoflurane and rapamycin treatment using two-way ANOVA with Bonferroni’s multiple comparisons test, rapamycin prevented the increase in pS6 labeling with 3.6% sevoflurane exposure (45.13 ± 8.77% for sevoflurane plus rapamycin compared to 39.42 ± 10.10% for rapamycin plus carrier gas, no significant difference) (Figure 6B). One adult MAC of sevoflurane is approximately 1.8% and, thus, compared to isoflurane, a higher dose of sevoflurane, which is at the high end of a clinically-reasonable concentration, is required to show an increase in pS6 expression.

Next, we tested the effects of propofol on pS6 expression. There was a significant increase in the percentage of pS6 positive cells measured in the 1nM propofol group (22.71 ± 5.46%, *p* < 0.0001), the 2 nM propofol group (23.96 ± 6.78%, *p* < 0.0001), and the 4 nM propofol group (29.02 ± 5.30%, *p* < 0.0001), compared to the control group (11.00 ± 6.52%) using one-way ANOVA with Dunnett’s multiple comparisons test (Figure 6C). Adding rapamycin 1 h before the 2nM propofol exposure decreased the pS6 immunoactivity (15.02 ± 2.63%) compared to the ones without rapamycin treatment (28.96 ± 3.78%, *p* < 0.01), and there was no significant difference between the 2 nM propofol + rapamycin group and the control + rapamycin group (13.29 ± 4.50%) using two-way ANOVA with Bonferroni’s multiple comparisons test, and there was a significant interaction between isoflurane and rapamycin treatment (Figure 6D). These data indicate that propofol may also mediate its effects though the mTOR pathway, although there is no clear way to draw equivalence in dosing between isoflurane or sevoflurane and propofol. One of the most practical strategies to potentially avoid anesthetic toxicity would be to choose drugs that do not activate pathways that result in toxic effects related to neural development. While numerous studies have identified mechanisms specific to either the potent volatile agents or to propofol [20], relatively few studies have conducted head-to-head comparisons between these two principal approaches to general anesthesia. Our data suggest to the extent that mTOR is a key mechanism in developmental anesthetic neurotoxicity, the choice of the agent may not be protective.

## 3. Materials and Methods

### 3.1. Neuronal Cultures

Primary neuron cultures were obtained from BrainBits, LLC (Springfield, IL, USA). Cultures consisted of dissociated neurons obtained from neocortex dissected from E18 Sprague Dawley rat embryos according to the company protocols. Neurons were plated on 12 mm glass coverslips at 16,000 cells/cm^2^ and maintained in NbActiv4 medium (BrainBits, Springfield, IL, USA) with half media changes conducted three times per week. Pilot experiments showed over 95% of cells from these cultures were immunopositive for β-tubulin, suggesting a high degree of purity. Experiments were performed on neurons between 3 and 14 DIV, and all experiments incorporated coverslips cultured from a minimum of three individual litters of pups.

### 3.2. Anesthetic Agent Exposure

Coverslips in 12-well plates were placed in identical air-tight, humidified chambers (Billups-Rothenberg, Del Mar, CA, USA) as previously described [43]. Isoflurane (Baxter Healthcare Cooperation, Deerfield, IL, USA) or sevoflurane (AbbVie Inc., North Chicago, IL, USA) were delivered using an agent-specific, calibrated inline vaporizer (SuperaVet, Vaporizer Sales and Services Inc., Rockmart, GA, USA), and were diluted in 5% CO_2_/95% O_2_ carrier gas. Controls for these experiments received 5% CO_2_/95% O_2_ carrier gas only. There was a 15-min equilibration period, which was required to achieve the correct concentration of isoflurane or sevoflurane as measured by a 5250 RGM gas analyzer (Datex-Ohmeda, Madison, WI, USA). Then the sealed chambers were placed in an incubator to maintain temperature at 37 °C for the duration of anesthesia exposure. Isoflurane/sevoflurane concentration was periodically measured at the end of the experimental period to verify that it was appropriately maintained throughout the exposure.

The propofol exposure was done by adding pure 2,6-diisopropylphenol (Sigma Aldrich, Saint Louis, MO, USA) (1 nM, 2 nM, 4 nM) into experiment wells, and incubated at 37 °C for the duration of anesthesia exposure. The exposure was terminated by removing all the media and by adding a combination of previously-removed media without propofol and fresh media.

### 3.3. The mTOR Pathway Inhibition

The mTOR inhibitors used in this study were as follows: PP242 at 1 μM (EMD Millipore, Billerica, MA, USA), and rapamycin at 100 nM (Sigma Aldrich, Saint Louis, MO, USA). They were used to inhibit mTORC1 or mTORC2, which are distinct functional pathways of the mTOR pathway. The same volume of the vehicle (DMSO) was added to the control groups. The neurons were pretreated with inhibitors 1 h before isoflurane or carrier gas exposure. The inhibitor concentration was maintained until the time of fixation by incorporating inhibitor in media changes.

### 3.4. TUNEL Assay

After isoflurane exposure, cells were harvested either immediately after the exposure or 24 h later. Neurons on coverslips were briefly fixed with 4% paraformaldehyde at room temperature for 10 min, then permeabilized and blocked for 1 h at room temperature in 5% donkey serum with 0.1% Triton X-100. Apoptosis was detected using an in situ cell death detection kit (Roche, Mannheim, Germany) and neurons were mounted on coverslips using 2.5% PVA/DABCO Mounting Media. An apoptotic index (AI) was defined as the number of TUNEL positive cells per field (400×) under a Leica SP8 confocal microscope (Leica, Wetzlar, Germany).

### 3.5. Immunocytochemistry

Fluorescent immunocytochemistry and labeling with fluorescently tagged F-actin was conducted as previously described [44]. Neurons on coverslips were briefly fixed with 4% paraformaldehyde at room temperature for 10 min, then permeabilized and blocked for 1 h at room temperature in 5% donkey serum with 0.1% Triton X-100. Neurons were incubated overnight at 4 °C in using the following antibodies: rabbit-anti-Synapsin-1 (1:200, EMD Millipore, Burlington, MA, USA), chicken-anti-Homer-1 (1:400, Synaptic Systems, Goettingen, Germany), mouse-anti-MAP-2 (1:200, Abcam, Cambridge, MA, USA), rabbit anti-human phospho-p70S6K (Thr-389, 1:1000, EMD Millipore, Billrecia, MA, USA), rabbit anti-human phospho-AKT (Ser-473,1:500, Cell Signaling Technologies, Danvers, MA, USA), rabbit anti-human S6 (1:100, Cell Signaling Technologies, Danvers, MA, USA), rabbit anti-human phospho-S6 (Ser-235/236, Cell Signaling Technologies, Danvers, MA, USA), and chicken-anti-human anti-β-III Tubulin (Tuj 1, 1:1000, EMD Millipore, Billrecia, MA, USA). All the antibodies were diluted in phosphate-buffered saline solution containing 0.1% Triton X-100. After rinsing, neurons were incubated for 2 h with a fluorescent secondary antibody and 4′,6-diamidino-2-phenylindole (DAPI) at the manufacturer’s recommended concentration (Jackson Immuno Research Labs, West Grove, PA, USA). Subsequently, neurons were mounted on coverslips using 2.5% PVA/DABCO mounting medium.

### 3.6. Imaging and Microscopic Analysis

A Leica SP8 confocal microscope was used to capture all microscopic images. Cell counting analyses were conducted manually. Excluding the coverslips for TUNEL assay, the counting field for each coverslip was conducted by capturing five 63× fields that were selected to represent all four quadrants and the center of the coverslip. Neuronal cell bodies were identified as those positive for both β-III Tubulin and DAPI, and representative images were taken using a 63× 1.0 N.A. objective with an additional 1.0× magnification lens in line. For the synaptic marker analysis, five neurons from each sample were evenly distributed throughout the coverslip to represent all four quadrants and the center was randomly selected for analysis. Images were taken using a 63× 1.0 N.A. objective with an additional 5× magnification lens in line. One dendrite was picked according to MAP-2 staining from each neuron and the locations for image taken were defined as 20–30 μm from the nucleus according to DAPI. Synaptic puncta were quantified using ImageJ software (NIH, Bethesda, MD, USA). The dendrite segment outline was traced and the area quantification was done according to the MAP-2 channel, and the threshold was maintained the same for the synaptic marker channel. The intensity of Synapsin-1/Homer-1 puncta inside the dendrite outline was measured and recorded. For TUNEL assay, neuronal cell bodies were identified as those positive for DAPI, and representative images were taken using a 40× 1.0 N.A. objective with an additional 1.0× magnification lens in line. The counting field for each coverslip was conducted by capturing five 40× fields that were selected to represent all four quadrants and the center of the coverslip. Both imaging and analysis were conducted by an investigator blind to the conditions.

### 3.7. Statistical Analysis

Results are expressed as mean ± SEM. All statistical analysis was conducted using Prism 6.0 (GraphPad, San Diego, CA, USA). Student’s *t*-test was used to determining statistical differences between each experiment group and the control-group data. One-way ANOVA with Dunnett’s multiple comparisons was used for the data with group number over two. Two-way ANOVA with Bonferroni’s multiple comparisons were used between groups that have the same exposure condition but different inhibitor treatments. All data examined with parametric tests were determined to be normally distributed and were conducted by an investigator blind to the conditions. Statistical significance for all tests were set a priori at *p* < 0.05.

## 4. Conclusions

In summary, we conclude that the potent volatile anesthetics and propofol, which are the mainstays of nearly all pediatric anesthetics, all have the capacity to up-regulate signaling in both branches of the mTOR pathway, mTOR1 and mTOR2, in neurons during synaptogenesis. Anesthetic exposure in this setting inhibits synaptogenesis, and this effect is reversible with the mTOR inhibitor, rapamycin. Our study has several limitations, principally related to the study of neural development in culture, where there is no patterned activity. In addition, because manipulation of mTOR via genetic means is problematic, only pharmacologic inhibition was used. Nevertheless, we believe that future studies of mTOR as a putative mechanism for developmental anesthetic neurotoxicity in dissociated culture will prove informative, and that questions about which types of neurons and synapses are at risk and what the effects on neural function are could be successfully addressed in this model system.

## Abbreviations

GA	General anesthetics
mTOR	Mechanistic target of rapamycin
mTORC1	mTOR 1 complex
mTORC2	mTOR 2 complex
DIV	Days in vitro
DAPI	4′,6-diamidino-2-phenylindole
DMSO	Dimethyl sulfoxide
pS6	Phosphorylated S6
MAC	Minimum alveolar concentration
Ser473	Ser473 phosphorylated Akt
Thr389	Thr389 phosphorylated 70S6
